# The association between community age-friendly facility diversity, types, leisure activities and frailty in Chinese older adults

**DOI:** 10.3389/fpubh.2025.1718744

**Published:** 2026-01-12

**Authors:** Zhanpeng Li

**Affiliations:** College of Art and Design, Nanjing Forestry University, Nanjing, China

**Keywords:** community age-friendly facilities, diversity, frailty, leisure activities, older adults

## Abstract

**Objective:**

Against the backdrop of a progressively aging population, frailty among older adults in China has become a major societal concern. While existing research has substantiated the association between community environment and health, studies examining the relationship from the perspective of the diversity and typology of age-friendly community facilities remain relatively scarce.

**Methods:**

Drawing on the 2023 China Longitudinal Aging Social Survey (CLASS) data, this study selected a sample of 10,453 older adults and employed structural equation modeling to analyze the correlations among the diversity and types of age-friendly community facilities, and the frailty of older adults.

**Results:**

The findings reveal a significant positive correlation between the community age-friendly facility diversity and frailty among older adults, which is largely mediated by leisure activity participation. Further analysis indicates differential associations between specific facility types and frailty: communities with senior libraries, communal dining halls, and health and wellness service centers are associated with significantly lower levels of frailty, whereas senior activity rooms are correlated with increased frailty. Moreover, the community age-friendly facility diversity, along with the presence of senior activity rooms, libraries, and communal dining halls, shows significant associations with frailty only among the younger-old subgroup. In contrast, community health service centers are negatively associated with frailty across all older adults. The mediating effect of leisure activities diminishes with advancing age.

**Conclusion:**

Therefore, future community age-friendly facility planning should not only focus on enhancing facility richness but also scientifically align facility types with the actual health needs and behavioral characteristics of older adults, thereby better supporting the implementation of China’s healthy aging strategy.

## 1 Introduction

Against the backdrop of an accelerating global aging process, developing countries—particularly China—are confronting profound social challenges arising from the rapid growth of the older adult population (1). By the end of 2024, China’s population aged 60 and above had exceeded 310 million. With advancing age, the gradual decline in physical function among older adults has drawn increasing attention from both society and academia (2, 3). Current research on factors influencing frailty of older adults has largely focused on conventional perspectives such as socioeconomic factors, living arrangements, and health behaviors, yet lacks innovative and feasible new approaches. Further investigation is still needed to clarify the determinants of frailty in late life (4, 5). Studies that systematically examine the role of community facilities in this context remain particularly scarce. Therefore, within the context of a deepening aging trend, how to mitigate frailty among older adults through optimizing age-friendly community facilities has become a pressing research priority.

Within the context of advancing the national strategy for active aging and the comprehensive implementation of community age-friendly renovation policies, the association between the age-friendly facilities in communities—the core setting for older adults’ daily activities—and the risk of frailty among this population has emerged as a critical issue in public health and policy-making (6, 7). Extensive research has confirmed that community environmental factors, such as green space levels and spatial accessibility, can reduce frailty risk by improving older adults’ physiological and psychological states (8–10). However, the current lack of ‘age-friendliness’ in community infrastructure development has made the optimal allocation of age-friendly facilities a key focus of livelihood policies at all levels of government (11).

While a small body of studies has preliminarily indicated that research into community facilities and residents’ health centers primarily on the practical value of individual facility types, existing findings are markedly misaligned with the needs of macro-level governmental policy-making. Some scholars have examined how the equity and accessibility of recreational facilities safeguard health and well-being of residents (12–14). Other studies have demonstrated that accessible healthcare facilities can improve health through timely interventions (15, 16), while public sports facilities can mitigate risks of obesity and frailty by promoting physical activity (17). Further research has elucidated the mechanisms through which single facilities affect health, such as recreational facilities alleviating frailty by enhancing social interaction and cognitive stimulation (18, 19). The value of such single-facility studies lies in providing direct evidence for the operational management and localized improvement of specific facilities, aligning with the practical needs of grassroots administrative departments (20). However, from the perspective of macro-level governmental policy formulation, studies focusing on single facility types exhibit significant limitations. Policy-makers are tasked with orchestrating the overall layout, typological mix, and resource allocation of community facilities, rather than concentrating solely on optimizing any single category. Existing research lacks systematic exploration of multidimensional attributes, such as facility quantity and type diversity, and consequently fails to address the core policy question of “how to configure combinations of facility types to prevent and control frailty risk among older adults” (21, 22).

As research in this field advances, scholars have come to recognize that the relationship between facility environments and the health is not merely direct, but involves complex mediating pathways (19, 23, 24). Socio-ecological theory provides a crucial framework for understanding this mechanism, emphasizing that the environment, individual behavior, and frailty constitute a dynamic interactive system that collectively shapes the health ecology of older adults (25). From this perspective, community-based age-friendly facilities do not only impact frailty directly; they also exert indirect effects by promoting behavioral engagement among older adults. Among these behaviors, leisure activities—as high-frequency components of daily life—hold particular significance as a key mediator (26, 27). Facilities such as activity centers, libraries, and health service centers help reduce environmental barriers to participation by providing safe, accessible, and suitable spaces and resources, thereby enhancing residents’ willingness and frequency of engagement in leisure activities (28, 29). Regular leisure activities, in turn, yield multiple physiological benefits: they improve muscle strength and flexibility, enhance balance and reduce the risk of falls (30), promote cardiovascular function and metabolism, aid in weight control and chronic disease management (31), and help maintain joint mobility and bone density, thereby slowing the decline of physical function (32). This pathway—from “facility environment → leisure activity → health improvement”—highlights the central role of leisure activities as a behavioral mediator. They serve not only as the crucial link converting environmental resources into health outcomes, but also as a behavioral expression of older adults’ active role in constructing their own health (33). Although the importance of such mediating mechanisms has gained preliminary recognition, existing studies still lack systematic investigation into the specific role of leisure activities in the facility–frailty relationship, and empirical analyses based on large-sample data remain particularly scarce.

Among older adult populations of different age groups, health status exhibits significant heterogeneity—a divergence that warrants particular attention in understanding the mechanisms through which the built environment influences health. Existing studies indicate that adults aged 75 and above demonstrate significantly higher levels of frailty, prevalence of chronic diseases, and mobility limitations compared to their younger-old counterparts, underscoring the pivotal role of age as a key demographic variable in the process of health decline. Concurrently, built environment factors—such as green space accessibility, air quality, and thermal comfort—have been highlighted in a number of empirical studies as influential to the physical and mental health of older adults across age subgroups, reflecting this population’s heightened sensitivity to changes in environmental conditions. Nevertheless, despite the growing attention to environmental health effects within the architectural and planning disciplines, research on how facility environments differentially impact frailty levels across distinct age cohorts of older adults remains notably scarce. There is still a lack of systematic investigation from the perspectives of diversity in facility offerings and stratification by facility types, often leaving policy to rely on empirical decision-making (7). More critically, the absence of studies examining the relationship between community age-friendly facility and frailty among diverse subgroups of older adults makes it difficult for policymakers to accurately align facility provision with the varied needs of the aging population. This gap may not only lead to inefficient allocation of facility resources but also undermine the potential of facility environments to effectively support healthy aging.

In summary, existing research on the relationship between community age-friendly facilities and frailty of older adults remains insufficient, with a particular scarcity of exploration into the underlying mediating mechanisms. To address the current challenge in China’s community age-friendly facility provision—the mismatch between supply and health demands—it is imperative to evaluate the impact of different facility types on frailty of older adults. In light of this, the present study utilizes data from the 2023 China Longitudinal Aging Social Survey (CLASS) and employs structural equation modeling to analyze the associations between the diversity of community age-friendly facilities, different facility types, and frailty of older adults, with a specific focus on examining the mediating role of leisure activities. The findings will help identify the facility types most strongly associated with health promotion, thereby providing an empirical basis for the targeted development of age-friendly communities under resource constraints and supporting the implementation of public policies oriented toward healthy aging. Specifically, the objectives of our study are as follows:

1. To identify the associations among the diversity and types of community age-friendly facilities and frailty among older adults.
2. To examine the mediating role of leisure activities in the associations between community age-friendly facilities and frailty.
3. To compare the differences in the associations between community age-friendly facilities and frailty across different age groups.
4. To propose differentiated allocation strategies for community age-friendly facilities tailored to different age groups.

## 2 Materials and methods

### 2.1 Data and samples

The data for this study were derived from the China Longitudinal Aging Social Survey (CLASS), a nationwide project led by Renmin University of China. Employing a multistage stratified probability sampling design, the survey covers 28 provinces/autonomous regions/municipalities across China, ensuring national representativeness. It aims to collect multidimensional data on health, environmental contexts of older adults through continuous longitudinal follow-ups. This study utilizes the 2023 wave of CLASS data. The sampling procedure consisted of three stages: first, random selection of villages/neighborhood committees as primary sampling units; second, random selection of households within the sampled villages/neighborhoods; and finally, random selection of one adult aged 60 or older from each household using the Kish grid method for face-to-face interviews. This process yielded an initial valid sample of 11,670 individuals.

To ensure data quality and mitigate potential bias caused by missing values, this study adopted a dual data processing strategy. First, during the preliminary processing stage, listwise deletion was applied to remove 1,405 samples with incomplete key variable information, resulting in an initial analytical sample of 10,265 individuals (sample retention rate: 88%). Second, considering that listwise deletion may introduce bias when missing data violate the Missing at Random (MAR) or Missing Not at Random (MNAR) assumptions, the study further employed multiple imputation (MI) for supplementary validation. All analyzed independent variables, dependent variables, and covariates were simultaneously included in the same imputation model, with the number of imputation iterations set to 30, generating 30 complete datasets. The analysis results from each imputed dataset were then pooled using Rubin’s rules, allowing a systematic comparison of the core statistical outcomes between the listwise deletion and multiple imputation approaches. Validation confirmed that the main research conclusions remained substantively consistent across both data processing methods, with key coefficients maintaining consistent directions and stable significance levels, indicating robust findings and demonstrating that missing value handling did not significantly affect the core inferences. Basic sample characteristics were as follows: 5,286 males (51%) and 4,979 females (49.0%); age range 61–103 years; educational attainment predominantly at the primary and junior high school levels; Han Chinese accounting for 93%. The sample structure meets the fundamental requirements for research on aging and health.

### 2.2 Variables and measurements

#### 2.2.1 Dependent variable: frailty

Frailty was assessed using the Instrumental Activities of Daily Living (IADL) scale recommended by the World Health Organization (34, 35). The scale comprises eight items: climbing stairs, walking outdoors, using transportation, shopping, managing finances, lifting heavy objects, housework, and cooking. Each item was rated on a 3-point scale (1 = “fully independent,” 2 = “partially dependent,” 3 = “completely dependent”), with total scores ranging from 8 to 24. Higher scores indicate greater levels of physical disability. The scale demonstrated good reliability and validity, with a Cronbach’s *α* coefficient of 0.910 and a KMO value of 0.848 (*p* < 0.001).

#### 2.2.2 Independent variable: community age-friendly facilities

The independent variable, community age-friendly facilities, is operationalized along two dimensions: facility diversity and facility type. Informed by the WHO’s Global Age-friendly Cities Guide and existing research strategies on community facilities, this study selects seven types of facilities closely related to the daily lives of older adults for measurement: senior activity rooms, senior fitness centers, chess/card rooms, senior libraries, outdoor activity areas, community dining halls, and health care service centers (36–39). Respondents answered the question “Does your community have the following facilities?” (0 = no, 1 = yes). Facility diversity was calculated as the summative index of the presence of these seven facility types, with higher scores indicating greater diversity of age-friendly facilities in the community.

#### 2.2.3 Mediating variables: leisure activities

Leisure activities served as the mediating variable, measured through participation frequency in four types of activities over the past year: square dancing, playing mahjong/chess, singing/playing musical instruments, and attending university for older adults or training courses (40). Each item was rated on a 5-point frequency scale (0 = “did not participate,” 1 = “a few times per year,” 2 = “at least monthly,” 3 = “at least weekly,” 4 = “almost daily”). Higher scores indicate more frequent engagement in leisure activities. The scale demonstrated acceptable reliability, with a Cronbach’s *α* coefficient of 0.782 and a KMO value of 0.815 (*p* < 0.001).

#### 2.2.4 Control variables

To address potential confounding factors in analyzing the association between community age-friendly facilities and frailty among older adults, this study incorporated multiple control variables to enhance the reliability and robustness of the findings. Educational level was coded on a 7-point scale (1 = illiteracy to 7 = postgraduate or above); gender as 1 = male and 2 = female; ethnicity as 1 = Han, 2 = other; economic status as 1 = relatively good, 2 = average, 3 = relatively poor; self-rated health on a 5-point scale (1 = very healthy to 5 = very unhealthy); perceived health change as 1 = improved, 2 = unchanged, 3 = worsened; BMI calculated as weight in kilograms divided by height in meters squared; hypertension, heart disease, diabetes, lumbar spine disease, arthritis, osteoporosis, smoking, and health insurance all coded as 1 = yes and 2 = no; household registration as 1 = urban and 2 = rural; and marital status as 1 = married and 2 = other. These covariates were systematically controlled to minimize confounding effects in the analytical model (41).

### 2.3 Research analysis

This study employed structural equation modeling (SEM) to analyze the complex relationships between community age-friendly facilities, leisure activities, and frailty among older adults. The SEM approach was adopted based on four key advantages: First, it effectively addresses measurement challenges associated with latent variables. Both leisure activities and frailty are multidimensional constructs; SEM integrates multiple observed indicators through confirmatory factor analysis, capturing the essential characteristics of these variables while isolating measurement error, thereby enhancing construct reliability and validity (42). Second, it enables simultaneous examination of direct and indirect pathways. SEM can concurrently estimate both the direct effects of community age-friendly facilities on frailty and the indirect mediating effects through leisure activities, systematically revealing the underlying mechanisms among variables (43). Third, it provides comprehensive model fit assessment. Through fit indices such as RMSEA and CFI, SEM allows objective evaluation of the correspondence between the theoretical model and empirical data, supports comparison across different models, and facilitates selection of the optimal theoretical framework, overcoming limitations of traditional regression methods (44). Fourth, it appropriately accommodates complex sampling design characteristics. Given that the CLASS database employed a multi-stage stratified sampling design, resulting in clustered data at the community level, we applied robust standard error estimation methods and specified communities as cluster variables. This approach addresses intra-class correlation by adjusting standard errors, ensuring accurate parameter estimation and valid statistical testing, thereby yielding more reliable inferential conclusions (45).

To assess the suitability of the data for SEM, we performed t-tests comparing high and low groups of all observed variables, split at the 27th and 73th percentiles. The results indicated significant differences between the high and low groups across all variables, confirming adequate discriminative power. Given that the dependent variable (IADL) was measured on a 3-point Likert scale, the mediating variable (LA) on a 5-point Likert scale, and the independent variables included both dichotomous and count variables, we employed the WLSMV (Weighted Least Squares Mean and Variance Adjusted) estimator in Mplus. This method is specifically designed for ordinal and non-continuous variables and provides robust parameter estimates and standard errors (45). During the analytical process, we first examined the measurement models for the two latent constructs—leisure activities and frailty. Confirmatory factor analysis demonstrated that the composite reliability (CR) values for the latent variables were 0.786 and 0.927, respectively, both exceeding the acceptable threshold of 0.7. The standardized factor loadings and squared multiple correlations (SMC) of the observed indicators also met or approached the recommended thresholds of 0.6 and 0.36 (46), indicating satisfactory reliability and validity of the measurement model and supporting the appropriateness of proceeding with structural equation modeling.

In this study, to control the increased error risk resulting from multiple hypothesis testing, we applied multiple comparison corrections to the statistical significance of all path coefficients. The hypothesis testing conducted on all key pathways could lead to an inflated family-wise error rate. To address this, we employed two widely used correction methods: false discovery rate control and family-wise error rate control (47). First, we implemented the Benjamini-Hochberg procedure to control the false discovery rate. This method does not strictly require all null hypotheses to be true, but rather controls the expected proportion of incorrect rejections among all rejected hypotheses, making it suitable for exploratory research contexts. Second, we simultaneously applied the more conservative Holm-Bonferroni correction to control the family-wise error rate. This method ensures that the probability of at least one false positive occurring when completing a set of tests does not exceed a predetermined threshold by progressively adjusting significance levels. All original *p*-values were ranked in ascending order, and corrected p-values were calculated according to the algorithms of both methods. Ultimately, we used a corrected *p*-value < 0.05 as the criterion for determining statistical significance. This dual-correction strategy not only ensures the reliability of the results but also maintains appropriate statistical power while strictly controlling false positives.

Given the cross-sectional nature of this study, propensity score matching (PSM) was implemented to supplement the structural equation modeling analysis. To better elucidate the relationship between community age-friendly facilities and frailty among older adults, PSM adjusts for potential confounding effects by systematically matching samples between comparison groups. This approach controls for confounding variables through propensity scores, thereby strengthening causal inferences regarding the associations. The PSM methodology operates by calculating propensity scores through binary logistic regression based on observed covariates, followed by one-to-one matching of cases across different groups. To ensure robustness, our analysis employed two distinct matching algorithms: nearest-neighbor matching and radius matching with a caliper size of 0.1.

Overall model fit indices showed that RMSEA and SRMR was below 0.08, and both CFI and TLI exceeded 0.90. All indices met established standards (44), indicating a good fit between the theoretical model and the data, and supporting its use for hypothesis testing (Table 1). All analyses were conducted using Mplus 8.0.

**Table 1:** Comparison of model fit metrics.

Model fit metrics	Idealized standards	Full sample model	95% CI	
			Lower	Upper
TLI	>0.900	0.931	–	–
CFI	>0.900	0.949	–	–
RMSEA	<0.080	0.055	0.048	0.063
SRMR	<0.080	0.048	0.036	0.058

## 3 Results

### 3.1 Descriptive statistics of research variables

Descriptive statistics of the study variables (Table 2) reveal that the mean value for the number of community age-friendly facilities is 1.909, indicating that Chinese communities are equipped with fewer than two such facilities on average (out of a total of seven types). This reflects the overall inadequacy in the development of age-friendly facilities in current Chinese communities. Regarding specific facility types, outdoor activity areas and senior activity rooms are relatively more common, whereas community dining halls and senior fitness centers show the lowest coverage rates. In terms of frailty among older adults, all observed variables scored above 1 point, suggesting a widespread presence of functional limitations to some extent. Particularly weaker performance was observed in activities such as lifting heavy objects, using transportation, and shopping, indicating that older adults face greater challenges in managing more complex daily tasks. As for leisure activities, the average participation frequency for all types of leisure activities hovered around “several times a year.” Square dancing recorded the highest participation frequency, while playing mahjong/chess showed the lowest. This pattern generally reflects a positive inclination among older adults toward engaging in leisure activities.

**Table 2:** Variable descriptive statistics.

Variable names	Observed variables	Variable items	Mean scores
Community age-friendly facilities	Senior activity room	Does your community have a senior activity room?	0.432
	Senior fitness centers	Does your community have a senior fitness center?	0.124
	Chess and card room	Does your community have a card and chess room?	0.315
	Senior libraries	Does your community have a senior library?	0.238
	Outdoor activity spaces	Does your community have outdoor activity spaces?	0.558
	Community canteens	Does your community have a community canteen hall?	0.111
	Health care service centers	Does your community have a community health service center?	0.131
	Facility diversity	Composite measure of facility quantity	1.909
Leisure activities	LA1	How often do you participate in senior university or training courses?	1.123
	LA2	How often do you participate in singing/playing musical instruments?	1.428
	LA3	How often do you participate in playing mahjong/chess?	1.108
	LA4	How often do you participate in square dancing?	1.493
Frailty	FR1	Are you able to climb up and down stairs?	1.077
	FR2	Are you able to walk outdoors?	1.053
	FR3	Are you able to use public transportation (e.g., bus, subway) independently?	1.106
	FR4	Are you able to go shopping for daily necessities independently?	1.128
	FR5	Are you able to manage your own finances?	1.071
	FR6	Are you able to lift an object weighing 5 kilograms (approx. 10 jin)?	1.137
	FR7	Are you able to prepare your own meals?	1.054
	FR8	Are you able to perform your own housework?	1.074
Control variables	Gender	What is your gender?	1.491
	Educational level	What is your highest level of education attained?	3.079
	Age	What is your current age?	73.675
	Economic status	What is your average monthly income?	1.943
	Ethnicity	What is your ethnicity?	1.373
	Number of cohabitants,	With how many people do you currently live?	2.719
	Self-rated health	How would you describe your current overall health?	2.417
	Perceived health change	Compared to 1 year ago, how would you rate your health in general now?	2.155
	BMI	Height (cm)/Weight^2^ (kg)	23.168
	Hypertension	Have you ever been diagnosed with hypertension?	1.618
	Heart disease	Have you ever been diagnosed with heart disease?	1.827
	Diabetes	Have you ever been diagnosed with diabetes?	1.849
	Lumbar spine disease	Have you ever been diagnosed with lumbar spine disease?	1.781
	Arthritis	Have you ever been diagnosed with arthritis?	1.740
	Osteoporosis	Have you ever been diagnosed with osteoporosis?	1.875
	Smoking	Do you currently smoke?	1.727
	Health insurance	Are you enrolled in any health insurance scheme?	1.498
	Household registration	Is your household registration (hukou) classified as urban or rural?	1.428
	Marital status	What is your current marital status?	1.169

Regarding the control variables, the analysis reveals a nearly balanced gender distribution within samples, with a mean age ranging from 73 to 74 years. The average educational attainment falls between primary and junior high school levels, while ethnic composition is predominantly Han Chinese. Economic status exhibited a normal distribution centered around the average range. The mean number of co-residents per household ranged from 2 to 3 individuals. Self-rated health status was concentrated in the “relatively healthy” to “average” categories, though most participants perceived a general decline in their health conditions. The mean Body Mass Index (BMI) was calculated at 23.168, indicating generally normal weight parameters across the sample. Chronic conditions were prevalent, with over half of the participants diagnosed with hypertension, heart disease, diabetes, arthritis, or lumbar spine diseases. More than half of participants reported current smoking habits. Health insurance coverage was nearly universal among respondents. The overwhelming majority reported being married.

### 3.2 Analysis of fitting results of full sample models

**Figure 1:**
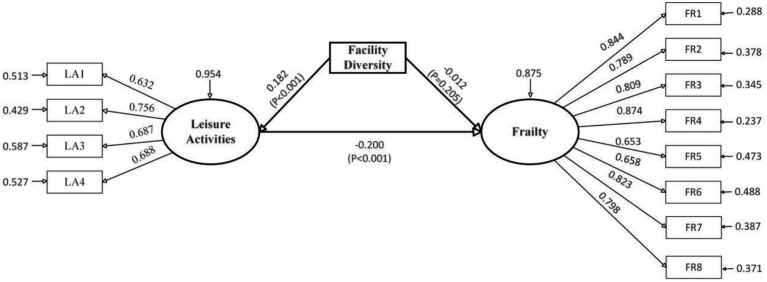
Standardization path diagram of the whole model. To simplify the visualization of the model, the effects of covariates in the model are hidden and the effect values are standardized coefficients.

Within the mediation analysis framework, the relationship between independent and dependent variables can be characterized through total, direct, and indirect effects. Table 3 and Figure 1 present the results of the full model incorporating mediating variables.

**Table 3:** Total, direct, and indirect effects of the full sample model.

Model path	Effect type	Effect estimate	SE	95% CI		*p*-value	FDR adjusted *p*-value
				Lower	Upper		
Facility diversity → Frailty	Total effect	−0.048	0.017	−0.091	−0.027	<0.001	<0.001
	Direct effect	−0.012	0.017	−0.053	−0.015	0.188	0.205
	Indirect effect	−0.036	0.011	−0.059	−0.015	<0.001	<0.001
Facility diversity → Leisure activities	Direct effect	0.182	0.021	0.092	0.253	<0.001	<0.001
Leisure activities → Frailty	Direct effect	−0.200	0.018	−0.253	−0.143	<0.001	<0.001
Gender	Direct effect	−0.050	0.012	−0.113	0.022	0.056	0.070
Age		0.184	0.010	0.160	0.207	<0.001	<0.001
Educational level		−0.034	0.011	−0.056	−0.011	0.002	0.004
Number of co-residents		0.103	0.009	0.083	0.123	<0.001	<0.001
Self-rated health		0.149	0.025	0.070	0.272	<0.001	<0.001
Perceived health change		0.304	0.025	0.231	0.441	<0.001	<0.001
Economic status		0.023	0.009	0.004	0.042	0.012	0.017
BMI		0.034	0.012	0.012	0.055	0.004	0.006
Hypertension		0.009	0.010	−0.009	0.028	0.342	0.373
Heart disease		0.069	0.010	0.046	0.094	<0.001	<0.001
Diabetes		0.036	0.010	0.016	−0.059	<0.001	<0.001
Lumbar spine disease		0.003	0.009	−0.015	0.021	0.783	0.817
Arthritis		0.024	0.010	0.004	0.044	0.013	0.017
Osteoporosis		−0.014	0.010	−0.034	0.006	0.150	0.171
Smoking		0.021	0.010	−0.013	0.042	0.064	0.076
Health insurance		−0.066	0.011	−0.089	−0.042	<0.001	<0.001
Household registration		0.009	0.024	−0.025	0.027	0.713	0.778
Ethnicity		0.066	0.009	0.041	0.095	<0.001	<0.001
Marital status		0.102	0.010	0.075	0.128	<0.001	<0.001

After controlling for demographic and socioeconomic variables, the diversity of community age-friendly facilities demonstrated a significant negative correlation with frailty among older adults. Specifically, only the total effect and indirect effect reached statistical significance, with coefficients of −0.048 and −0.037, respectively. This indicates that the correlation between facility diversity and reduced frailty was fully mediated by leisure activity participation among older adults. Furthermore, leisure activities showed a significant negative correlation with frailty (coefficient: −0.200), suggesting that higher frequency of leisure activities is associated with lower frailty levels. Finally, a significant positive correlation was observed between community age-friendly facility diversity and leisure activities (coefficient: 0.182), indicating that greater facility diversity is associated with increased frequency of leisure activity participation among older adults.

Regarding the control variables, age, number of co-residents, ethnicity, marital status, self-rated health, perceived health change, BMI, heart disease, diabetes, arthritis, and economic status all showed significant positive correlations with frailty among older adults. The effect size for age was 0.191, indicating that advanced age is associated with more severe frailty. The number of co-residents had an effect size of 0.081, suggesting that larger household size may be linked to poorer frailty outcomes. Ethnicity and marital status demonstrated effect sizes of 0.066 and 0.102, respectively, revealing that Han Chinese ethnicity and being married are associated with better physical condition. Self-rated health and perceived health change exhibited effect sizes of 0.173 and 0.118, reflecting a reasonable consistency between older adults’ subjective health perceptions and their actual health status. BMI showed an effect size of 0.038, indicating that higher BMI values correlate with relatively worse frailty conditions. The presence of heart disease, diabetes, and arthritis was associated with increased frailty, with effect sizes of 0.069, 0.036, and 0.024, respectively. Economic status demonstrated an effect size of 0.023, indicating that poorer economic conditions are related to more pronounced frailty. In contrast, educational level and health insurance coverage displayed significant negative correlations with frailty, with effect sizes of −0.034 and −0.066, respectively, demonstrating that lower educational attainment and lack of health insurance are associated with worse frailty outcomes. These findings align with empirical observations and existing research literature, thereby supporting the reliability of the study’s results.

### 3.3 Model results of different types of community age-friendly facilities and frailty of older adults

**Figure 2:**
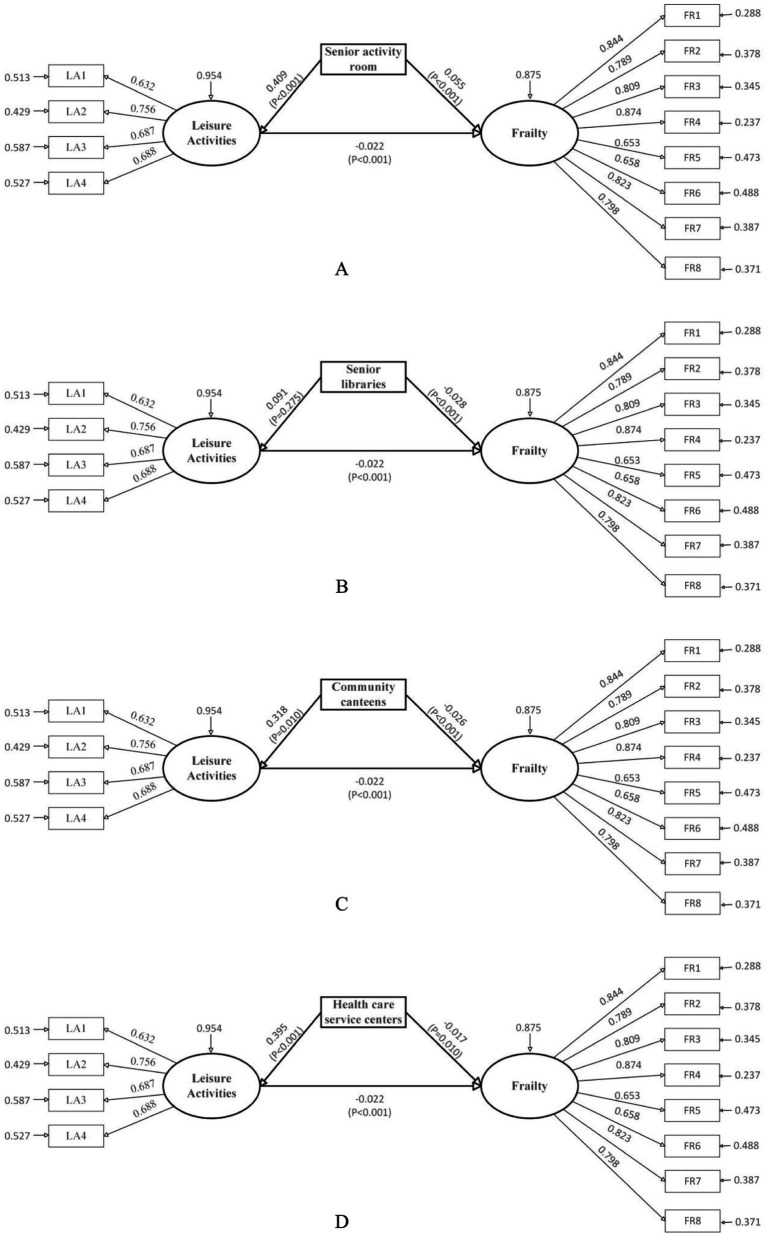
Model results of different types of facilities and frailty of older adults. To simplify the visualization of the model, the effects of covariates in the model are hidden and the effect values are standardized coefficients. **(A)** Senior activity room. **(B)** Senior libraries. **(C)** Community canteens. **(D)** Healthcare service centers.

To further investigate the associations between specific types of community age-friendly facilities and the frailty of older adults, this study constructed separate analytical models with different facility types as independent variables (see Table 4; Figure 2).

**Table 4:** The overall effect, direct effect and indirect effect of different types of community age-friendly facilities and frailty of older adults.

Model path	Effect type	Effect estimate	SE	95% CI		*p*-value	FDR adjusted *p*-value
				Lower	Upper		
Senior activity room	Total effect	0.046	0.014	0.019	0.078	<0.001	<0.001
	Direct effect	0.055	0.014	0.027	0.082	<0.001	<0.001
	Indirect effect	−0.009	0.004	−0.021	−0.003	<0.001	<0.001
Senior fitness centers	Total effect	0.005	0.011	−0.016	0.028	0.656	0.826
	Direct effect	0.015	0.011	−0.005	0.038	0.166	0.249
	Indirect effect	−0.010	0.003	−0.018	−0.004	<0.001	<0.001
Chess and card room	Total effect	0.009	0.014	−0.022	0.031	0.635	0.826
	Direct effect	0.016	0.014	−0.013	0.037	0.235	0.329
	Indirect effect	−0.007	0.003	−0.015	−0.004	0.008	0.014
Senior libraries	Total effect	−0.030	0.012	−0.054	−0.005	<0.001	<0.001
	Direct effect	−0.028	0.012	−0.052	−0.001	<0.001	<0.001
	Indirect effect	−0.002	0.001	−0.006	0.000	0.147	0.231
Outdoor activity spaces	Total effect	−0.005	0.015	−0.033	0.025	0.753	0.826
	Direct effect	0.005	0.015	−0.023	0.035	0.716	0.826
	Indirect effect	−0.010	0.004	−0.017	−0.003	0.005	0.009
Community canteens	Total Effect	−0.033	0.010	−0.051	−0.013	<0.001	<0.001
	Direct Effect	−0.026	0.010	−0.045	−0.007	<0.001	<0.001
	Indirect effect	−0.007	0.002	−0.013	−0.002	0.010	0.017
Health care service centers	Total effect	−0.026	0.012	−0.049	−0.007	0.005	0.009
	Direct effect	−0.017	0.012	−0.040	−0.005	0.010	0.017
	Indirect effect	−0.009	0.003	−0.015	−0.003	0.003	0.009
Leisure activities → Frailty	Direct effect	−0.022	0.011	−0.043	−0.014	<0.001	<0.001

The results indicate that senior libraries, community dining halls, and community health care service centers demonstrated statistically significant negative associations with frailty (total effects: *β* = −0.030, −0.033, and −0.026, respectively). Conversely, senior activity rooms showed a significant positive association with frailty (total effect: *β* = 0.046). No significant associations were observed for senior fitness centers, chess/card rooms, or outdoor activity areas.

Collectively, the results demonstrate substantial heterogeneity in how different facility types correlate with frailty, distinguishing them from the aggregate effect of general facility diversity. Specifically, both community dining halls and community senior service centers showed significant direct and indirect effects on frailty among older adults, with direct effect values of −0.026 and −0.017, and indirect effect values of −0.007 and −0.008, respectively. This indicates that these two facility types are associated with reduced frailty levels partly through promoting participation in leisure activities among older adults. In contrast, senior libraries demonstrated a significant direct effect on frailty (effect value: −0.028) but no significant indirect effect, suggesting that libraries primarily mitigate frailty through their inherent functions rather than through leisure activity mediation. On the other hand, senior activity rooms showed a direct effect of 0.055 and an indirect effect of −0.009 on frailty. Notably, their total and direct effects were positive, indicating that the presence of senior activity rooms is significantly associated with poorer frailty outcomes, while leisure activities partially mediate this relationship and attenuate the facility’s potential negative association.

### 3.4 Robustness checks

To ensure the robustness of the research findings, three distinct validation methods were employed, all of which consistently supported the statistical results and conclusions of the model, demonstrating strong reliability:

1. The study employed propensity score matching (PSM) to validate the structural equation modeling results. Samples were divided into treatment (top 50% in facility indicators) and control groups. Using key demographic variables, propensity scores were calculated via binary logit model. Nearest-neighbor matching achieved 100% success rate with standardized biases below 20% and non-significant t-tests (*p* > 0.05). The association between age-friendly facilities and frailty remained significant, confirming the robustness of main conclusions.
2. Reduction of control variables: When only core demographic variables—age, gender, education level, and number of cohabitants—were retained, the direction of influence coefficients remained consistent with the original model, confirming the stability of the conclusions.
3. Bootstrap sampling method: Using 1,000 bootstrap samples to compute parameter distributions, standard errors, and confidence intervals, the model estimates were found to be highly consistent with the original results.

### 3.5 Heterogeneity analysis

Given the varying physical conditions among different social groups, it is crucial to examine the pathway differences in the “community age-friendly facilities → leisure activities → frailty” model across older adult subgroups. Using multi-group structural equation modeling, we compared path coefficients and variable means across groups to precisely identify intergroup variations. Groups were first stratified by gender, age, and urban–rural residence. With metric invariance constrained, results showed only the age-group model exhibited significant pathway differences (*p* < 0.05), whereas gender and residence models demonstrated no significant disparities (*p* > 0.05).

#### 3.5.1 Facility diversity and frailty across age groups

Regarding community age-friendly facilities diversity, significant associations with frailty were observed exclusively among younger-old adults. Both the total effect (−0.059) and indirect effect (−0.043) of facility availability on frailty reached statistical significance in this group, while the direct effect was non-significant. This indicates that the association between facility availability and reduced frailty operates predominantly through promoting leisure activity participation among younger-old adults.

#### 3.5.2 Facility types and frailty across age groups

Regarding specific facility types, senior activity rooms, libraries, and community dining halls showed significant associations solely among younger-old adults. In contrast, community senior service centers demonstrated significant associations with frailty across all older adults, though these effects diminished with advancing age.

For senior activity rooms, the total, direct, and indirect effects on frailty among younger-old adults were 0.060, 0.066, and −0.006, respectively, indicating a positive association between the facilities and frailty, though leisure activities partially mitigated this adverse relationship. Regarding senior libraries, significant total and direct effects were observed (−0.055 and −0.054), with no significant indirect effect, suggesting that the protective association operates entirely through the libraries’ inherent functions rather than through leisure activity mediation. Community dining halls demonstrated significant total, direct, and indirect effects of −0.044, −0.040, and −0.004, respectively, confirming their negative association with frailty in younger-old adults, with leisure activities serving a minor mediating role. The negative association between community health service centers and frailty diminished with advancing age, showing total effects of −0.030, −0.027, and −0.021 for younger-old, middle-old, and older-old adults, respectively; direct effects of −0.020, −0.021, and −0.010; and indirect effects of −0.010, −0.006, and −0.011 (Table 5).

**Table 5:** Statistical values of the correlation between the diversity and types of community age-friendly and frailty of the older adults in different age groups.

Model path	Effect type	Effect estimate	SE	95% CI		*p*-value	FDR adjusted *p*-value
				Lower	Upper		
Facility diversity (younger-old adults)	Total effect	−0.059	0.018	−0.085	−0.015	<0.001	<0.001
	Direct effect	−0.016	0.019	−0.028	−0.005	0.025	0.041
	Indirect effect	−0.043	0.008	−0.075	−0.016	<0.001	<0.001
Senior activity room (younger-old adults)	Total effect	0.060	0.025	0.031	0.097	<0.001	<0.001
	Direct effect	0.066	0.026	0.037	0.096	<0.001	<0.001
	Indirect effect	−0.006	0.004	−0.013	−0.002	0.033	0.052
Senior libraries (younger-old adults)	Total effect	−0.055	0.017	−0.089	−0.021	<0.001	<0.001
	Direct effect	−0.054	0.017	−0.085	−0.025	<0.001	<0.001
	Indirect effect	−0.001	0.001	−0.003	0.005	0.229	0.321
Community canteens (younger-old adults)	Total effect	−0.044	0.027	−0.079	−0.018	<0.001	<0.001
	Direct effect	−0.040	0.021	−0.075	−0.023	<0.001	<0.001
	Indirect effect	−0.004	0.006	−0.010	−0.001	0.040	0.060
Health care service centers (younger-old adults)	Total effect	−0.030	0.019	−0.053	−0.009	<0.001	<0.001
	Direct effect	−0.020	0.017	−0.035	−0.011	<0.001	<0.001
	Indirect effect	−0.010	0.004	−0.016	−0.003	0.008	0.015
Health care service centers (middle-old adults)	Total effect	−0.027	0.017	−0.049	−0.006	0.010	0.018
	Direct effect	−0.021	0.016	−0.041	−0.003	<0.001	<0.001
	Indirect effect	−0.006	0.004	−0.012	−0.006	0.015	0.026
Health care service centers (older-old adults)	Total effect	−0.021	0.020	−0.042	−0.002	0.024	0.040
	Direct effect	−0.010	0.019	−0.021	−0.001	0.035	0.054
	Indirect effect	−0.011	0.009	−0.021	−0.007	0.008	0.015

## 4 Discussion

Drawing on the CLASS 2023 dataset, this study employed structural equation modeling to establish a “community age-friendly facilities → leisure activities → frailty of older adults” theoretical framework. It systematically examined the pathways linking both the diversity and specific types of community age-friendly facilities with the frailty of older adults, while testing the mediating role of leisure activities. The findings contribute to identifying critical facility types that support healthy aging and provide empirical evidence for applying socio-ecological theory to older populations.

### 4.1 Association between community age-friendly facility diversity and frailty in older adults

This study elucidates the correlation between the diversity of community age-friendly facilities and frailty among older adults, along with its underlying mechanism. First, the research identifies a direct and significant protective effect of facility diversity against frailty. This finding substantiates and extends previous studies on community environment and health (11–13), clearly demonstrating that beyond mere availability, facility diversity constitutes a critical factor. A community environment rich in facilities provides diverse activity options and enhances mobility convenience, thereby helping delay the decline of muscle strength and physical function. Moreover, the study reveals the crucial mediating role of leisure activities in this relationship. This indicates that age-friendly facilities not only produce direct effects but also indirectly mitigate frailty by engagement in leisure activities. This “facilities-behavior-health” pathway delineates a potential chain: diverse age-friendly facilities reduce barriers and costs for activity participation, thereby promoting regular physical activity and positive social interaction, collectively contributing to the retardation of frailty progression (48).

### 4.2 Variation in associations between facility types and frailty in older adults

Following detailed classification of age-friendly facility types, this study reveals significant variations in the pathways through which different facilities associate with frailty, providing nuanced insights for targeted resource allocation.

Specifically, senior libraries demonstrate a complete direct association with frailty reduction without significant mediation through leisure activities. This suggests library benefits may derive not from promoting physical activities but through more immediate mechanisms. As hubs of health information, reading materials may directly empower older adults by stimulating cognitive function, alleviating psychological stress, and enhancing health literacy and self-management capabilities, thereby directly contributing to frailty mitigation (49).

In contrast, community dining halls and health and wellness service centers exhibit a partial mediation pattern with both direct and indirect pathways. These facilities provide immediate services (e.g., nutritional meals, basic health monitoring) that directly alleviate frailty. More importantly, they function as vital social hubs within communities. Dining halls serve not only as eating venues but also as informal social centers where interpersonal interactions may help maintain physical function. Wellness centers systematically promote physical activity and social participation through organized cultural events and health lectures, drawing older adults from private homes to community spaces, thereby indirectly mitigating frailty progression (50, 51).

Notably, senior activity rooms demonstrate a complex association pattern: a positive direct association with frailty alongside a negative indirect association through leisure activities. This reflects two coexisting mechanisms—selection and compensation. The positive direct association may indicate health-based selection: as highly accessible community spaces, these rooms naturally attract frailer individuals, while healthier seniors opt for alternative venues, creating a baseline negative total association (52–54). However, the negative indirect association reveals a compensatory mechanism: for those already experiencing frailty, these spaces provide protective benefits through social and activity engagement. This finding highlights the need to both enhance facilities’ compensatory functions through improved programming and address selective participation patterns through integrated resource planning.

### 4.3 Age-stratified associations between community age-friendly facilities and frailty

This study reveals a noteworthy pattern: both the diversity of age-friendly facilities and specific types demonstrate significant associations with frailty solely among the younger-old cohort. This finding underscores an “age-graded” effect in environment-health relationships. Younger-old adults, typically retaining greater functional capacity and health literacy, are better positioned to proactively utilize environmental resources (55). Community age-friendly facilities provide diverse options that encourage behaviors like participating in community activities, and socializing in dining halls, thereby helping maintain physical function. However, for older-old adults, accumulating health complications may create an invisible “health threshold” that substantially limits their ability and willingness to actively use these universal facilities. Consequently, facility “diversity” does not necessarily translate into individual “accessibility,” with potential health benefits being overshadowed by physiological aging. This suggests that while universal design effectively serves active younger-old adults, it may inadvertently marginalize the most vulnerable older-old population who need support most.

Unlike other facilities, community health service centers demonstrate a consistent negative association with frailty across all age groups, though the magnitude of this overall effect diminishes with advancing age. This indicates a paradigm shift in how these centers function: from “behavioral promotion” to “direct care.” For younger-old adults, these centers may facilitate healthy lifestyles through organized health education and social interactions, representing a preventive approach. As individuals advance into older age, the core value of health services shifts from prevention and promotion to management and maintenance (56). Here, the direct, professional services—chronic disease monitoring, rehabilitation guidance, medication consultation—become crucial, operating through more immediate pathways. Thus, the attenuated mediation effect reflects not functional failure but an adaptive response to the most pressing needs of the oldest-old: transitioning from supporting “how to live well” to enabling “how to live well with conditions,” precisely illustrating the indispensable safety-net role of community health services in late life.

### 4.4 Implications and limitations

Building on the core findings, this study proposes three feasible measures for community age-friendly facility planning: First, establishing a mechanism for universal facility optimization and health-oriented renewal. For facilities demonstrating significant functional associations like senior libraries and community dining halls, their capacity to promote active leisure engagement should be enhanced through targeted design interventions—such as incorporating health literacy zones and specialized lecture spaces in libraries, and implementing nutritionally-balanced menus with communal dining programs in cafeterias. Particularly crucial is implementing functionally-stratified renovations for senior activity rooms: creating designated zones for passive socialization, light physical activities, and structured exercises according to varying functional capacities, complemented by professional guidance from rehabilitation specialists. This transforms activity rooms from recreational spaces into multi-functional health-promoting environments through professionally curated programming.

Second, adopting an age-stratified facility allocation and targeted service strategy. Planning frameworks should clearly distinguish between “vitality-enhancing facilities” targeting younger-old adults and “support-oriented facilities” serving older-old populations. New communities should prioritize installing fitness trails and multi-purpose activity spaces, while existing neighborhoods incorporate corresponding functional modules through micro-regeneration projects. Simultaneously, implementing a community health navigation system with professional assessment of older-old adults’ needs would provide personalized facility utilization plans and assistance services, addressing accessibility barriers resulting from functional decline.

Third, developing a hub-and-spoke model with health service centers as community cores. Positioning these centers as central nodes in community health networks would leverage their age-universal protective associations through dual pathways: extending preventive health programming into various facilities for younger-old adults, while establishing integrated care teams (physicians, nurses, aging specialists) to provide direct care coordination and professional referrals for older-old adults, thereby creating a proactive support network addressing diverse health needs.

This study has several limitations that warrant attention in future research. First, the cross-sectional design using CLASS 2023 data precludes causal inference, establishing only correlational relationships between facility environments and health of older adults. Future investigations should employ longitudinal data to examine potential causal pathways. Second, although representing the most recent CLASS survey, the 2023 dataset reflects conditions from 2 years prior, potentially not fully capturing current dynamics in China’s age-friendly facilities and health status of older adults. Ongoing analysis with updated datasets is recommended. Third, the study lacks granular objective measures of facility characteristics, including accessibility and density metrics. Subsequent research would benefit from examining how such specific environmental factors correlate with frailty progression in older populations. Finally, while this study, based on a large sample, has validated a significant association between community age-friendly facilities and frailty in older adults through leisure activity engagement, the standardized coefficients for key pathways in the model remain relatively modest. This suggests that the independent direct effect of any single environmental factor is limited, and that these “statistically significant” associations warrant cautious evaluation in terms of real-world policy intervention effectiveness. Future research should incorporate a broader theoretical framework, integrating additional moderating or mediating variables at the individual, social, and environmental levels, to more comprehensively explain the mechanisms through which age-friendly facilities influence frailty. Such efforts would help explore integrated intervention strategies capable of yielding greater health benefits.

## 5 Conclusion

This study reveals that the overall diversity of age-friendly facilities in Chinese communities remains limited, while facility diversity demonstrates a significant negative correlation with frailty among older adults. Specifically, communities with greater facility diversity generally show better health outcomes among older adults compared to those with scarce facilities. More importantly, distinct types of age-friendly facilities show varying associations with frailty of older adults. Notably, different types of age-friendly facilities exhibit varying correlations with frailty. The presence of senior libraries, community dining halls, and senior service centers correlates with better frailty outcomes, whereas senior activity rooms show a significant negative correlation with frailty levels. Leisure activities consistently function as a mediating factor in these relationships, indicating that facilities correlate with health status through their association with behavioral patterns among older adults. Furthermore, the study identifies age-stratified patterns: senior activity rooms, libraries, and community dining halls demonstrate significant correlations exclusively with frailty in younger-old adults. In contrast, community health service centers maintain significant correlations with frailty across all age groups, though these correlations diminish with advancing age. Therefore, in advancing community age-friendly initiatives, beyond merely increasing facility quantity, greater emphasis should be placed on functional diversity and selectivity, with priority given to configuring facility types that demonstrate clear health-promoting correlations. This approach will not only contribute to improving daily quality of life and frailty outcomes among older adults but also provide substantive support for promoting active aging.

## Data Availability

The raw data supporting the conclusions of this article will be made available by the authors, without undue reservation.

## References

[1] Wang H Chen H. Aging in China: challenges and opportunities. China CDC weekly. (2022) 4:601. doi: 10.46234/ccdcw2022.130 35919296 PMC9339359

[2] Yan Y Du Y Li X Ping W Chang Y. Physical function, ADL, and depressive symptoms in Chinese elderly: evidence from the CHARLS. Front Public Health. (2023) 11:1017689. doi: 10.3389/fpubh.2023.1017689, 36923048 PMC10010774

[3] Lin H Wan M Ye Y Zheng G. Effects of Baduanjin exercise on the physical function of middle-aged and elderly people: a systematic review and meta-analysis of randomized controlled trials. BMC Complement Med Ther. (2023) 23:38. doi: 10.1186/s12906-023-03866-4, 36747221 PMC9901146

[4] Kim GM Hong MS Noh W. Factors affecting the health-related quality of life in community-dwelling elderly people. Public Health Nurs. (2018) 35:482–9. doi: 10.1111/phn.12530, 29947059

[5] Zhang Y Su D Chen Y Tan M Chen X. Effect of socioeconomic status on the physical and mental health of the elderly: the mediating effect of social participation. BMC Public Health. (2022) 22:605. doi: 10.1186/s12889-022-13062-7, 35351078 PMC8962021

[6] Li X Krumholz HM Yip W Cheng KK De Maeseneer J Meng Q . Quality of primary health care in China: challenges and recommendations. Lancet. (2020) 395:1802–12. doi: 10.1016/S0140-6736(20)30122-7, 32505251 PMC7272159

[7] Xiang L Shen GQ Tan Y Liu X. Emerging evolution trends of studies on age-friendly cities and communities: a scientometric review. Ageing Soc. (2021) 41:2814–44. doi: 10.1017/S0144686X20000562

[8] Weimann A Kabane N Jooste T Hawkridge A Smit W Oni T. Health through human settlements: investigating policymakers’ perceptions of human settlement action for population health improvement in urban South Africa. Habitat Int. (2020) 102203. doi: 10.1016/j.habitatint.2020.102203

[9] Hu Q Wang C. Quality evaluation and division of regional types of rural human settlements in China. Habitat Int. (2020) 105:102278. doi: 10.1016/j.habitatint.2020.102278

[10] Zheng Z Chen L Wang Y Jin Y Liu Y. Habitat for health: exploring the impact of rural human settlements on elderly behaviors and physical function across gender in Sichuan, China. J Asian Archit Build Eng. (2025):1–17. doi: 10.1080/13467581.2025.2487262

[11] Salmistu S Kotval Z. Spatial interventions and built environment features in developing age-friendly communities from the perspective of urban planning and design. Cities. (2023) 141:104417. doi: 10.1016/j.cities.2023.104417

[12] Chen Q Zhang Z Mao Y Deng R Shui Y Wang K . Investigating the influence of age-friendly community infrastructure facilities on the health of the elderly in China. Buildings. (2023) 13:341. doi: 10.3390/buildings13020341

[13] Zhang Z Qiu Z. The usage pattern and spatial preference of community facilities by elder people in rural environments. J Housing Built Environ. (2019) 35:661–78. doi: 10.1007/s10901-019-09707-6

[14] Wang Y Jin C Lu M Lu Y. Assessing the suitability of regional human settlements environment from a different preferences perspective: a case study of Zhejiang Province, China. Habitat Int. (2017) 70:1–12. doi: 10.1016/j.habitatint.2017.09.010

[15] Bu T Tang D. Transportation infrastructure and good health in urban China. Hum Soc Sci Commun. (2025) 12:1–18. doi: 10.1057/s41599-025-05060-y, 39310270

[16] Yin C He Q Liu Y Chen W Gao Y. Inequality of public health and its role in spatial accessibility to medical facilities in China. Appl Geogr. (2018) 92:50–62. doi: 10.1016/j.apgeog.2018.01.011

[17] Fang CY Chen PY Liao Y. Factors influencing seniors' willingness to pay intention for exercise in the civil sports and recreation centers. Front Public Health. (2023) 10:992500. doi: 10.3389/fpubh.2022.992500, 36777771 PMC9911538

[18] Chen Y Liu B Shen Y Li L. Assessing accessibility to service facilities for older people in age-restricted communities from the perspective of equity. J Transp Health. (2022) 27:101515. doi: 10.1016/j.jth.2022.101515

[19] Guida C Carpentieri G Masoumi H. Measuring spatial accessibility to urban services for older adults: an application to healthcare facilities in Milan. Eur Transp Res Rev. (2022) 14:23. doi: 10.1186/s12544-022-00544-3, 38625259 PMC9160510

[20] Rutter H Savona N Glonti K Bibby J Cummins S Finegood DT . The need for a complex systems model of evidence for public health. Lancet. (2017) 390:2602–4. doi: 10.1016/S0140-6736(17)31267-9, 28622953

[21] Chu Y Zhang H. Do age-friendly community policy efforts matter in China? An analysis based on five-year developmental plan for population aging. Int J Environ Res Public Health. (2022) 19:13551. doi: 10.3390/ijerph192013551, 36294133 PMC9603113

[22] Ellen ME Panisset U de Carvalho IA Goodwin J Beard J. A knowledge translation framework on ageing and health. Health Policy. (2017) 121:282–91. doi: 10.1016/j.healthpol.2016.12.009, 28108136

[23] Amankwah O Choong WW Boakye-Agyeman NA. The relationship between facilities management service quality and patients’ health-care experience: the mediating effect of adequacy of health-care resource. Facilities. (2023) 41:108–25. doi: 10.1108/F-08-2022-0113

[24] Amankwah O Weng-Wai C Mohammed AH. Modelling the mediating effect of health care healing environment on core health care delivery and patient satisfaction in Ghana. Environ Health Insights. (2019) 13:1178630219852115. doi: 10.1177/1178630219852115, 31217690 PMC6560799

[25] Stokols D. Establishing and maintaining healthy environments: toward a social ecology of health promotion. Am Psychol. (1992) 47:6–22. doi: 10.1037/0003-066X.47.1.6, 1539925

[26] Crandon TJ Scott JG Charlson FJ Thomas HJ. A social–ecological perspective on climate anxiety in children and adolescents. Nat Clim Chang. (2022) 12:123–31. doi: 10.1038/s41558-021-01251-y

[27] Yueh KY Chang WJ. Leisure activity participation among older adults: a review. Leis Stud. (2024) 44:1–20. doi: 10.1080/02614367.2024.2353604

[28] Tao Y Zhang W Gou Z Jiang B Qi Y. Planning walkable neighborhoods for “aging in place”: lessons from five aging-friendly districts in Singapore. Sustainability. (2021) 13:1742. doi: 10.3390/su13041742

[29] Khoddam H Dehghan M Sohrabi A Modanloo M. The age–friendly cities characteristics from the viewpoint of elderly. J Family Med Prim Care. (2020) 9:5745–51. doi: 10.4103/jfmpc.jfmpc_1098_20, 33532425 PMC7842487

[30] Caban-Martinez AJ Courtney TK Chang WR Lombardi DA Huang YH Brennan MJ . Leisure-time physical activity, falls, and fall injuries in middle-aged adults. Am J Prev Med. (2015) 49:888–901. doi: 10.1016/j.amepre.2015.05.022, 26232899

[31] Cheng W Zhang Z Cheng W Yang C Diao L Liu W. Associations of leisure-time physical activity with cardiovascular mortality: a systematic review and meta-analysis of 44 prospective cohort studies. Eur J Prev Cardiol. (2018) 25:1864–72. doi: 10.1177/2047487318795194, 30157685

[32] Young CM Weeks BK Beck BR. Simple, novel physical activity maintains proximal femur bone mineral density, and improves muscle strength and balance in sedentary, postmenopausal Caucasian women. Osteoporos Int. (2007) 18:1379–87. doi: 10.1007/s00198-007-0400-6, 17572834

[33] Cai J Hu T Zhou L Jiang H Gao Y. Effects of leisure activities and general health on the survival of older people: a cohort study in China. Front Public Health. (2023) 11:1273074. doi: 10.3389/fpubh.2023.1273074, 37854240 PMC10579939

[34] Beltz S Gloystein S Litschko T Laag S van den Berg N. Multivariate analysis of independent determinants of ADL/IADL and quality of life in the elderly. BMC Geriatr. (2022) 22:894. doi: 10.1186/s12877-022-03621-3, 36418975 PMC9682836

[35] Chatterji S Byles J Cutler D Seeman T Verdes E. Health, functioning, and disability in older adults—present status and future implications. Lancet. (2015) 385:563–75. doi: 10.1016/S0140-6736(14)61462-8, 25468158 PMC4882096

[36] World Health Organization. Measuring the age-friendliness of cities: A guide to using core indicators. Geneva, Switzerland: World Health Organization (2015).

[37] Jiang C Chow JCC Zhou L Song H Shi J. Community support, social isolation and older adults’ life satisfaction: evidence from a national survey in China. Aging Ment Health. (2024) 28:849–57. doi: 10.1080/13607863.2023.2277871, 37921357

[38] Mu Y Yi M Liu Q. Association of neighborhood recreational facilities and depressive symptoms among Chinese older adults. BMC Geriatr. (2023) 23:667. doi: 10.1186/s12877-023-04369-0, 37848820 PMC10583466

[39] Yan S Jiang S Dong X Guo X Chen M. Public sports facility availability in living communities and mental health of older people in China: the mediating effect of physical activity and life satisfaction. Behavioral Sciences. (2025) 15:991. doi: 10.3390/bs15070991, 40723775 PMC12292078

[40] Jopp DS Hertzog C. Assessing adult leisure activities: an extension of a self-report activity questionnaire. Psychol Assess. (2010) 22:108–20. doi: 10.1037/a0017662, 20230157 PMC2841313

[41] Bernerth JB Aguinis H. A critical review and best-practice recommendations for control variable usage. Pers Psychol. (2016) 69:229–83. doi: 10.1111/peps.12103

[42] Hellier PK Geursen GM Carr RA Rickard JA. Customer repurchase intention: a general structural equation model. Eur J Mark. (2003) 37:1762–800. doi: 10.1108/03090560310495456

[43] Fornell LDF. Evaluating structural equation models with unobservable variables and measurement error. J Mark Res. (1981) 18:39–50.

[44] Mulaik SA James LR Van Alstine J Bennett N Lind S Stilwell CD. Evaluation of goodness-of-fit indices for structural equation models. Psychol Bull. (1989) 105:430. doi: 10.1037/0033-2909.105.3.430

[45] Cureton EE. The upper and lower twenty-seven per cent rule. Psychometrika. (1957) 22:293–6. doi: 10.1007/BF02289130

[46] Raykov T Shrout PE. Reliability of scales with general structure: point and interval estimation using a structural equation modeling approach. Struct Equ Model Multidiscip J. (2002) 9:195–212. doi: 10.1207/S15328007SEM0902_3

[47] Li A Barber RF. Multiple testing with the structure-adaptive Benjamini–Hochberg algorithm. J R Stat Soc Ser B Stat Methodol. (2019) 81:45–74. doi: 10.1111/rssb.12298

[48] Miralles-Guasch C Dopico J Delclòs-Alió X Knobel P Marquet O Maneja-Zaragoza R . Natural landscape, infrastructure, and health: the physical activity implications of urban green space composition among the elderly. Int J Environ Res Public Health. (2019) 16:3986. doi: 10.3390/ijerph16203986, 31635362 PMC6843616

[49] Luyt B Ann HS. Reading, the library, and the elderly: a Singapore case study. J Libr Inf Sci. (2011) 43:204–12. doi: 10.1177/0961000611418813

[50] Wang X Liu M Li Y Guo C Yeh CH. Community canteen services for the rural elderly: determining impacts on general mental health, nutritional status, satisfaction with life, and social capital. BMC Public Health. (2020) 20:230. doi: 10.1186/s12889-020-8305-9, 32059652 PMC7023764

[51] Cho YI Lee SYD Arozullah AM Crittenden KS. Effects of health literacy on health status and health service utilization amongst the elderly. Soc Sci Med. (2008) 66:1809–16. doi: 10.1016/j.socscimed.2008.01.003, 18295949

[52] Oliveira JS Gilbert S Pinheiro MB Tiedemann A Macedo LB Maia L . Effect of sport on health in people aged 60 years and older: a systematic review with meta-analysis. Br J Sports Med. (2023) 57:230–6. doi: 10.1136/bjsports-2022-105820, 36450439 PMC9933166

[53] Van Cauwenberg J Nathan A Barnett A Barnett DW Cerin E Council on Environment and Physical Activity (CEPA)-Older Adults Working Group. Relationships between neighbourhood physical environmental attributes and older adults’ leisure-time physical activity: a systematic review and meta-analysis. Sports Med. (2018) 48:1635–60. doi: 10.1007/s40279-018-0917-1 29721838

[54] Ali MJ Rahaman M Hossain SI. Urban green spaces for elderly human health: a planning model for healthy city living. Land Use Policy. (2022) 114:105970. doi: 10.1016/j.landusepol.2021.105970

[55] Liu S Ho HC. Effects of socioeconomic status and greenspace on respiratory emergency department visits under short-term temperature variations: an age-stratified case time-series study. Soc Sci Med. (2024) 343:116613. doi: 10.1016/j.socscimed.2024.116613, 38290398

[56] Abe T Carver A Sugiyama T. Associations of neighborhood built and social environments with frailty among mid-to-older aged Australian adults. Geriatr Gerontol Int. (2021) 21:893–9. doi: 10.1111/ggi.14253, 34355479

