# Supplementary material for: The association between community age-friendly facility diversity, types, leisure activities and frailty in Chinese older adults
Source: Front Public Health. 2026 Jan 12;13:1718744. doi: 10.3389/fpubh.2025.1718744 (PMC12833224; doi:10.3389/fpubh.2025.1718744)
Supplement: Supplementary file 1 [file Table_1.docx]

**Supplementary materials**

To avoid errors arising from the multiple testing burden, we simultaneously examined all facilities using multiple indicators within a single SEM. The findings align with the original results, with the key path significance remaining consistent, which demonstrates robust credibility. For details, please refer to Supplementary Table 1.

**Supplementary Table 1. The overall effect, direct effect and indirect effect of different types of community age-friendly facilities and physical function of older adults.**

| **Independent variables** | **Mediator variables** | **Dependent variable** | | |
| --- | --- | --- | --- | --- |
|  | **Leisure Activity** | **Physical Function** | | |
|  |  | **Total Effect** | **Direct Effect** | **Indirect Effect** |
| Senior activity room | 0.283*** | 0.046*** | 0.054*** | -0.008*** |
| Senior libraries | 0.052* | -0.024*** | -0.023*** | -0.001 |
| Community canteens | 0.132*** | -0.036*** | -0.032*** | -0.004** |
| Health care service centers | 0.178*** | -0.021** | -0.017** | -0.004** |
| Leisure activity | - | -0.027*** | -0.027*** | - |

Notes: *** means significance at the 0.001 confidence level;

** means significance at the 0.01 confidence level;

* means significance at the 0.05 confidence level.
